# Compliance With Antibiotic Prophylaxis in Hip Arthroplasty Following Neck of Femur Fractures: A Retrospective Clinical Audit Study

**DOI:** 10.7759/cureus.97941

**Published:** 2025-11-27

**Authors:** Mahmoud Mersal, Jija Thomas, Muhammad Shahab, Nayan Shrivastava, Mohamed Elgendy, Osama Embaby, Mohamed Elmarakby, Serajdin Ajnin

**Affiliations:** 1 Trauma and Orthopaedics, University Hospitals Birmingham NHS Foundation Trust, Birmingham, GBR; 2 Orthopaedics, Sandwell and West Birmingham Hospitals NHS Trust, Birmingham, GBR; 3 Orthopaedics, Mansoura University Hospital, Birmingham, GBR

**Keywords:** clinical audit, fracture neck of femur, hip arthroplasty, periprosthetic joint infection (pji), superficial skin infection

## Abstract

Background: Periprosthetic joint infection (PJI) and superficial wound or skin infection are serious complications following hip arthroplasty for neck of femur (NOF) fractures. To reduce these risks, perioperative antibiotic prophylaxis is recommended in national guidance, and our trust's local guideline specifies three postoperative intravenous doses of flucloxacillin or teicoplanin in patients with a penicillin allergy. This audit aimed to assess compliance with this protocol and its association with postoperative wound and skin infection rates.

Methodology: A retrospective audit study was conducted at Birmingham Heartlands Hospital, part of the University Hospitals Birmingham NHS Foundation Trust, United Kingdom (UK), between October 1 and November 30, 2023. All patients undergoing hip arthroplasty for NOF fractures were included. Data were extracted from the patient information and communication system (PICS), which records clinical notes, medication charts, and operative documentation. The audit standard was defined as 100% compliance with the administration of three postoperative intravenous doses of flucloxacillin or teicoplanin in penicillin-allergic patients, in accordance with the trust's guideline. Patients managed conservatively were excluded.

Results: A total of 179 patients were included (83 intracapsular, 95 extracapsular, and one periprosthetic fracture). Postoperative antibiotics were administered to 150 patients (83.8%), while 29 (16.2%) received none. Flucloxacillin was the most common agent (n=121), with 69.4% (84/121) completing all three recommended doses. Alternative antibiotics included teicoplanin (n=13) for penicillin allergy and co-amoxiclav (n=8), levofloxacin (n=4), clindamycin (n=2), and clarithromycin (n=2) for patients already on antibiotic therapy or with specific clinical indications. Three postoperative wound infections (1.7%) were recorded, two in patients without postoperative antibiotics and one in a teicoplanin-treated penicillin-allergic patient. No PJIs occurred.

Conclusion: Compliance with the trust's guideline was suboptimal, with only 84 of 121 flucloxacillin recipients (69.4%) completing all three postoperative doses. Superficial wound infections occurred in two patients who received no postoperative antibiotics and in one teicoplanin-treated case, and no PJIs were observed. Improved documentation, prescriber education, and routine monitoring are needed to optimise prophylaxis and sustain very low infection rates.

## Introduction

Hip arthroplasty for neck of femur (NOF) fractures is one of the most frequently performed operations in elderly patients, with approximately 75,000 cases annually in the United Kingdom (UK) [1]. Despite advances in surgical technique, perioperative optimisation, and aseptic precautions, surgical site infection (SSI), including both superficial skin infection and deep periprosthetic joint infection (PJI), remains a significant complication. Even minor superficial wound infections can delay healing, increase pain, prolong hospital stay, and in some cases, progress to deeper prosthetic infection associated with high morbidity, mortality, and healthcare cost [2-4].

The incidence of SSI following hip arthroplasty is reported to range between 1-3%, with PJI accounting for approximately 0.5-1% of cases [5,6]. Although less severe, superficial wound infections can precede deeper contamination of the prosthetic interface and therefore should not be underestimated [2,3]. Evidence consistently demonstrates that appropriate perioperative antibiotic prophylaxis substantially reduces the risk of both superficial and deep infections, with meta-analyses showing up to 80% reduction in SSI rates when prophylactic regimens are correctly timed and completed [7].

According to the National Institute for Health and Care Excellence (NICE) and the British Orthopaedic Association (BOA) Standards for Trauma (BOAST) hip fracture guidelines, patients undergoing hip arthroplasty for NOF fractures should receive perioperative antibiotic prophylaxis, with our trust's local guideline specifying three postoperative intravenous doses of flucloxacillin or teicoplanin in cases of a penicillin allergy [8-10]. This regimen provides sustained bactericidal coverage during the critical early postoperative period when wound colonisation risk is highest.

Despite clear recommendations, compliance with antibiotic prophylaxis protocols in practice is often inconsistent. Missed or incomplete doses, handover lapses, lack of electronic prescribing prompts, and documentation omissions remain recurrent causes of deviation from the guidelines. These lapses can compromise infection control and clinical outcomes.

This audit, therefore, aimed to assess compliance with the trust’s postoperative antibiotic prophylaxis guideline following hip arthroplasty for NOF fractures and evaluate the association between adherence and postoperative wound or skin infection rates, including the occurrence of PJIs.

## Materials and methods

A retrospective clinical audit was conducted at Birmingham Heartlands Hospital, part of the University Hospitals Birmingham NHS Foundation Trust, UK, and registered under the audit code CARMS-21016. The audit period spanned from October 1 to November 30, 2023.

All consecutive patients who underwent hip arthroplasty for an NOF fracture during the audit period were included. Data were retrieved from the patient information and communication system (PICS), which records real-time clinical documentation, operative notes, and electronic prescriptions. Additional details were verified from postoperative notes and discharge summaries where required.

Inclusion and exclusion criteria

Patients aged 18 years or older undergoing hip arthroplasty, hemiarthroplasty, or total hip replacement for intracapsular, extracapsular, or periprosthetic NOF fractures were included. Patients managed conservatively, those treated with internal fixation alone, and cases with incomplete operative documentation were excluded.

Audit standard

The audit standard was based on the trust's local guideline for antimicrobial prophylaxis in hip arthroplasty following NOF fractures [8]. This guideline specifies three postoperative intravenous doses of flucloxacillin (1 g every six hours) or teicoplanin in case of patients with documented penicillin allergy. These regimens form part of the wider hip fracture care pathway, which was developed with reference to NICE CG124 and BOA BOAST standards on hip fracture management [9,10]. The target compliance rate with the local antimicrobial prophylaxis guideline was defined as 100%.

Data collected

Variables included patient demographics, fracture type, operative procedure, postoperative antibiotic agent and dosing schedule, documentation quality, and any recorded superficial wound and skin infections or PJI during the index admission.

For patients not given postoperative antibiotics, the documented reason for omission was extracted verbatim from the operative and postoperative notes.

## Results

A total of 179 patients met the inclusion criteria, including 83 intracapsular, 95 extracapsular, and one periprosthetic NOF fracture. The overall patient distribution by fracture type is summarised in Table 1, which demonstrates an almost equal split between intracapsular and extracapsular fractures, with a single periprosthetic case recorded during the audit period.

**Table 1:** Fracture type distribution

Fracture type	n	% of total (n=179)
Intracapsular	83	46.40%
Extracapsular	95	53.10%
Periprosthetic	1	0.60%
Total	179	100%

Postoperative antibiotics were administered to 150 of 179 patients (83.8%), while 29 patients (16.2%) did not receive postoperative prophylaxis. The breakdown of antibiotic administration status is presented in Table 2, highlighting that although the majority received prophylaxis, a significant minority did not.

**Table 2:** Postoperative antibiotic administration

Status	n	% of total (n=179)
Antibiotics given	150	83.80%
No antibiotics given	29	16.20%
Total	179	100%

Among the 29 patients who did not receive postoperative antibiotics, documentation review revealed three main reasons. A total of 12 cases had antibiotics mentioned in the postoperative notes but not prescribed on the chart; eight were explicitly documented as “not required,” and nine had no mention of antibiotics in the operative or postoperative notes. Two patients within this group developed superficial wound infections, and no PJIs were reported. This breakdown is presented in Table 3.

**Table 3:** Documentation and reasons for omission

Reason recorded in notes	n	% of no antibiotic group
Mentioned in notes but not prescribed	12	41.40%
Explicitly recorded as “not required”	8	27.60%
Not mentioned in postoperative documentation	9	31.00%
Total	29	100%

Among the 150 patients who received postoperative antibiotics, flucloxacillin was the most commonly prescribed agent (121 patients, 80.7%). Of these, 84 patients (69.4%) received all three recommended doses, 28 (23.1%) received two doses, and nine (7.4%) received a single dose. These data are summarised in Table 4.

**Table 4:** Flucloxacillin dose completion The table summarises dosing compliance among patients prescribed flucloxacillin.

Doses completed	n	% of flucloxacillin group
3 doses	84	69.40%
2 doses	28	23.10%
1 dose	9	7.40%
Total	121	100%

Alternative postoperative antibiotics, as listed in Table 5, were prescribed only in specific clinical circumstances. Teicoplanin was administered to 13 patients (8.7%), all of whom had a documented penicillin allergy. In several of these cases, a single intraoperative dose of gentamicin was also given in accordance with the local perioperative infection control policy [8]. When patients who completed the full three-dose flucloxacillin regimen and those who received the recommended single dose of teicoplanin were considered together, 97 of 179 patients (54.2%) were fully compliant with the postoperative antibiotic guideline. 

**Table 5:** Alternative postoperative antibiotic regimens and indications LRTI, lower respiratory tract infection; CAP, community-acquired pneumonia; UTI, urinary tract infection

Antibiotic agent	n	Indication (as recorded in notes)
Teicoplanin	13	Penicillin allergy (all)
Co-amoxiclav	8	Pre-op infection: LRTI (3), CAP (2), sepsis of unknown origin (2), UTI (1)
Levofloxacin	4	Penicillin allergy (2); on treatment for chest infection (2)
Clindamycin	2	Pre-op infection: UTI (1), LRTI (1)
Clarithromycin	2	Pre-op infection: CAP (2)

Co-amoxiclav was prescribed to eight patients (5.3%) who were already receiving antibiotic therapy before surgery for active infections, including lower respiratory tract infection (n=3), community-acquired pneumonia (n=2), sepsis of unknown origin (n=2), and urinary tract infection (n=1). Clindamycin was given to two patients (1.3%), one being treated for a urinary tract infection and the other for a lower respiratory tract infection, before their operation. Clarithromycin was used in two patients (1.3%), both already on treatment for community-acquired pneumonia prior to surgery. Finally, levofloxacin was prescribed to four patients (2.7%), of whom two had penicillin allergy and two were already undergoing therapy for chest infection.

When antibiotic compliance was stratified by regimen, it became evident that patients who received the full three-dose flucloxacillin protocol had no postoperative infections, whereas incomplete or omitted antibiotic courses were associated with higher superficial wound infection rates. Three postoperative superficial wound or skin infections (1.7%) occurred: two in patients who received no postoperative antibiotics and one in a teicoplanin-treated penicillin-allergic patient. No infections were observed among patients who completed or partially completed the flucloxacillin regimen, or among those receiving other alternative agents. No cases of PJI were identified (Table 6).

**Table 6:** Antibiotic compliance and postoperative infection outcomes * Other alternative regimens include co-amoxiclav, levofloxacin, clindamycin, and clarithromycin, as described in Table 5.

Category	n	% of total (n=179)	Post-op infections (n)	Infection rate (%)
Flucloxacillin - 3 doses	84	46.90%	0	0.00%
Flucloxacillin - 1-2 doses	37	20.70%	0	0.00%
Teicoplanin (penicillin-allergic)	13	7.30%	1	7.70%
Other alternative regimens *	16	8.90%	0	0.00%
No postoperative antibiotics	29	16.20%	2	6.90%
Total	179	100%	3	1.70%

## Discussion

This audit demonstrated suboptimal compliance with the trust’s postoperative antibiotic prophylaxis guideline for hip arthroplasty following NOF fractures. Among patients prescribed flucloxacillin, only 69.4% completed the full three-dose regimen. Overall, 83.8% of patients received at least one postoperative antibiotic dose, yet incomplete dosing and inconsistent documentation remained significant areas for improvement.

The observed postoperative wound and superficial skin infection rate of 1.7% aligns with previously reported figures following hip arthroplasty, typically ranging between 1-3% [5,6]. Importantly, no infections occurred in patients who completed the full three-dose flucloxacillin regimen, reinforcing the importance of full adherence to the recommended prophylaxis protocol. The absence of PJI during the audit period is consistent with published evidence demonstrating that appropriate prophylaxis substantially reduces the risk of deep infections [3,7].

It is notable that the single postoperative infection in the teicoplanin-treated group (one of 13 patients, 7.7%) represents the highest infection proportion among the subgroups. Given the very small sample size, no causal inference can be drawn, and this finding is likely to reflect random variation and underlying patient or procedural risk factors rather than the choice of agent itself. Nevertheless, it underscores that adherence to penicillin allergy protocols alone does not eliminate infection risk and supports continued monitoring of outcomes in this subgroup in future audit cycles.

Superficial wound and skin infections, though less severe than PJI, remain clinically important because they may precede deeper contamination of the prosthetic interface [2,4]. Early bacterial colonisation of the surgical site, if not adequately suppressed by postoperative antibiotics, can serve as a precursor to PJI [3]. A large meta-analysis in hip fracture surgery has confirmed that appropriate perioperative antibiotic prophylaxis significantly reduces both superficial and deep SSIs compared to no prophylaxis [7]. In the elective arthroplasty setting, more recent systematic reviews and trials have not demonstrated a clear advantage of prolonged postoperative prophylaxis over shorter or single-dose regimens, and practice in many centres is moving toward limiting antibiotic duration [11,12]. Our audit did not seek to evaluate the optimal duration of prophylaxis but instead focused on adherence to the existing local policy, which specifies a three-dose postoperative regimen. These findings, together with World Health Organization (WHO) guidance, support the principle that whatever regimen is chosen, it should be applied consistently within a structured perioperative infection prevention pathway [13].

The reasons for noncompliance identified in this audit, including documentation errors, missing prescriptions, and unclear postoperative plans, mirror those reported in previous UK audits [9,10]. Such process failures are often related to human factors, particularly communication gaps between theatre and ward teams and variation in understanding of local antibiotic policies. Integrating electronic prescribing prompts within the PICS, together with targeted education for surgical, anaesthetic, and ward staff, may improve adherence and reduce the likelihood of missed postoperative doses.

Although the overall infection rate observed in this audit is low, even isolated cases of PJI carry serious implications. PJI is associated with prolonged hospital stay, repeated surgical procedures, extended courses of antibiotic therapy, and a substantial financial burden for the NHS [14,15]. Preventing such complications through rigorous antibiotic compliance and robust perioperative pathways is therefore both clinically essential and economically justified.

Study limitations

This audit has several limitations. Its retrospective design and reliance on existing documentation introduce the potential for incomplete data capture and misclassification. The relatively short audit period and the lack of long-term follow-up may underestimate the true incidence of infection, particularly late-presenting PJI. Furthermore, potentially relevant factors such as operative duration, surgical approach, body mass index, smoking status, and detailed comorbidity burden were not analysed, despite their recognised influence on infection risk. Nevertheless, the findings provide a realistic snapshot of local practice and a clear platform for targeted quality improvement.

Overall, this audit underscores the importance of consistent antibiotic administration, accurate documentation, and regular compliance monitoring. Implementing these measures is likely to further reduce superficial wound infections and help maintain the absence of PJI observed within this cohort.

## Conclusions

Compliance with postoperative antibiotic prophylaxis following hip arthroplasty for NOF fractures was below the desired standard, with only 69.4% of patients prescribed flucloxacillin completing the recommended three-dose regimen. All observed superficial wound infections occurred in patients who either received no postoperative antibiotics or belonged to the small teicoplanin-treated subgroup of penicillin-allergic patients, while no infections were seen in those who completed the full flucloxacillin protocol, and no PJIs were observed. Strengthening documentation, improving prescriber awareness, and integrating electronic safeguards within PICS are essential to enhance adherence, optimise patient safety, and maintain low infection rates.
